# Toward Precision Obesity Pharmacotherapy: Using the Eating Behavior Phenotype Scale (EFCA) in Real-World Clinical Practice

**DOI:** 10.3390/nu18091419

**Published:** 2026-04-30

**Authors:** Ronaldo José Pineda-Wieselberg, Andressa Heimbecher Soares, Thiago Fraga Napoli, Nilza Maria Scalissi, João Eduardo Nunes Salles

**Affiliations:** Discipline of Endocrinology, Santa Casa de Misericórdia de São Paulo, São Paulo 01221-020, Brazil; andressaendocrinologista@gmail.com (A.H.S.); napoli38@gmail.com (T.F.N.); nscalissi@uol.com.br (N.M.S.); jensalles@yahoo.com.br (J.E.N.S.)

**Keywords:** obesity, precision medicine, obesity phenotypes, anti-obesity medications, behavioral scales

## Abstract

Background: Obesity is a heterogeneous chronic disease in which eating behavior phenotypes may influence treatment response. Yet, anti-obesity medication (AOM) selection is still largely guided by anthropometric and metabolic parameters, with limited use of behavioral phenotyping in routine practice. We evaluated whether multidimensional eating behavior changes, measured by the Brazilian Eating Behavior Phenotype Scale (*Escala de Fenótipos do Comportamento Alimentar*, EFCA), differ across commonly used AOMs in a real-world cohort. Methods: We conducted a retrospective, observational, real-world study in obesity outpatient care settings in São Paulo, Brazil. Adults with obesity (18–65 years) treated with a single principal AOM for 6 months and paired baseline/6-month follow-up EFCA and anthropometric data were included. Analyses focused on early responders (≥5% total body weight loss at 3 months). Five AOM groups available in Brazil were analyzed: semaglutide (oral or subcutaneous), naltrexone/bupropion, sibutramine, topiramate, and tirzepatide. Outcomes included percent weight loss, EFCA total score, and five EFCA subscales (hedonic, emotional, compulsive, hyperphagic, disorganized). Within-medication behavioral changes were assessed using paired tests and standardized effect sizes (Cohen’s dz, 95% CI), summarized in heatmap form. Results: The analytical cohort comprised 66 early responders with paired EFCA assessments at baseline and 6 months. EFCA profiling revealed distinct behavioral response fingerprints across AOMs. Effect size mapping showed predominantly large behavioral effects (many dz ≥ 0.8) in hedonic, emotional, hyperphagic, and compulsive domains. Strongest signals included emotional eating reductions with naltrexone/bupropion (dz 2.04), tirzepatide (dz 1.77), semaglutide (dz 1.52), and topiramate (dz 1.54); hedonic reductions with tirzepatide (dz 2.06), semaglutide (dz 1.55), and naltrexone/bupropion (dz 1.52); hyperphagic reductions with tirzepatide (dz 1.50) and semaglutide (dz 1.34); and compulsive reductions with topiramate (dz 1.41) and consistent effects across tirzepatide, semaglutide, and sibutramine (≈dz 0.95–0.96). Disorganized eating showed heterogeneous/attenuated responsiveness, from near-null with tirzepatide (dz 0.03) to large but imprecise effects in smaller groups (e.g., topiramate dz 1.24, wide CI). Conclusions: In this responder-enriched real-world cohort, AOMs showed distinct and reproducible EFCA behavioral signatures, supporting a clinically actionable phenotype-informed framework to prioritize, sequence, and monitor obesity pharmacotherapy beyond nonspecific weight reduction, while highlighting disorganization as a potential target for adjunctive behavioral strategies.

## 1. Introduction

Obesity is a complex, chronic, and multifactorial disease resulting from the interaction between genetic susceptibility, environmental factors, metabolic regulation, and eating behavior. While excess adiposity is traditionally attributed to energy imbalance, growing evidence indicates that heterogeneity in eating behavior phenotypes plays a central role in obesity onset, maintenance, and treatment response. Therefore, uniform therapeutic strategies frequently lead to variable and unpredictable outcomes [1].

Pharmacological treatment of obesity has expanded substantially over the past decade, with agents targeting distinct neuroendocrine pathways involved in appetite regulation, reward processing, and impulse control [2]. However, response to anti-obesity medications remains highly heterogeneous, and currently available guidelines provide limited guidance on how to individualize pharmacotherapy beyond anthropometric or metabolic parameters [3], with the Brazilian 2026 guideline being the first to consider the use of eating patterns to guide medication use [4]. This highlights the need for tools capable of identifying behavioral phenotypes that may predict differential response to specific medications.

The Eating Behavior Phenotype Scale (*Escala de Fenótipos do Comportamento Alimentar*—EFCA) [5], also validated for the Brazilian Portuguese [6], was first developed to capture clinically relevant dimensions of eating behavior, including hedonic eating, compulsive eating, emotional eating, hyperphagia, and disorganized eating patterns. These domains reflect partially independent neurobehavioral mechanisms involving reward circuitry, executive control, emotional regulation, and satiety signaling. Previous studies have suggested that such behavioral phenotypes are associated with obesity severity and metabolic risk; however, their role in guiding pharmacological treatment selection remains insufficiently explored [7,8].

Despite this theoretical rationale, there is a lack of empirical data evaluating the differential effects across distinct eating behavior phenotypes, particularly using validated multidimensional behavioral scales and real-world clinical data. Moreover, most studies assessing pharmacological outcomes focus predominantly on weight loss, with limited attention to behavioral changes that may precede or accompany anthropometric responses. Previous initiatives that tried to use obesity phenotypes to guide pharmacological choice had success [9], but the process of phenotyping people with obesity was long and difficult to reproduce.

Therefore, the present study aimed to evaluate changes in EFCA total score and subscale domains before and after pharmacological treatment with anti-obesity medications (AOM) available in Brazil, and to investigate whether specific eating behavior phenotypes are associated with greater behavioral responsiveness to this medication. By integrating behavioral, anthropometric, and metabolic data, this study seeks to contribute to the growing field of phenotype-guided obesity pharmacotherapy, offering insights into a more personalized approach to obesity management.

## 2. Materials and Methods

### 2.1. General Objective

Evaluate retrospectively the answer to anti-obesity medications (AOM) according to clinical phenotype, using the Brazilian version of the EFCA [6].

### 2.2. Specific Objectives

Identify and categorize different clinical obesity phenotypes in a sample of early responders according to EFCA.

Analyze the correlation between clinical obesity phenotypes and AOM using early responders as a model to identify possible phenotypes for each AOM.

### 2.3. Methods

#### 2.3.1. Study Design and Setting

This was an observational, retrospective, and real-world study conducted at Santa Casa de São Paulo, in our outpatient service for obesity management in São Paulo, Brazil. Data came from individuals following in the obesity management ambulatory and in private practice, also in São Paulo, Brazil.

#### 2.3.2. Participants

We screened adult patients (aged between 18 and 65 years) with obesity who initiated pharmacotherapy for weight management and had standardized assessments both at baseline (pre-treatment) and at follow-up after 6 months of treatment. Inclusion criteria were: the clinical diagnosis of obesity (defined as BMI higher than 29.9 kg/m^2^ and/or BMI higher than 26.9 kg/m^2^, increased waist circumference accordingly to gender and South American cutoff (males > 90 cm and females > 80 cm) and waist to height relation higher than 0.5 [1]; availability of baseline and follow-up body weight; completion of the EFCA total score and subscales at both time points (baseline and six months); treatment with one of the five main pharmacological options analyzed and available in Brazil: subcutaneous and oral semaglutide [10,11,12], naltrexone/bupropion [13,14,15], sibutramine [16,17], topiramate [18], or tirzepatide [19], as the only anti-obesity medication during the six month interval; and being early responders to AOM, defined as weight loss higher than 5% of total body weight in three months [20]. Exclusion criteria were: age higher than 65 years old or below 18 years old; diagnosis of diabetes; secondary or syndromic causes of obesity, such as Cushing syndrome, insulinoma, lipodystrophy or Prader–Willi syndrome; high cardiovascular risk or higher; missing key identifiers preventing pairing of baseline–follow-up; pregnancy or lactation during follow-up; bariatric surgery or analog surgical conditions during the observation window; use of combination anti-obesity regimens that precluded attribution to a principal medication; and cognitive impairment to answer the EFCA. The analytical cohort comprised *n* = 66 unique individuals. The baseline of all individuals per AOM is presented in Table 1.

The rationale for considering only early responders to AOM in our sample is that these individuals lose more weight in comparison to non-early responders [21], but they also behave as a metabolic model of success; thus, the difference in their response could suggest what would work in non-early responders as well. Similar comparisons could be made by comparing individuals with type 1 diabetes as a model of insulinopenia, aiming to identify whether outcome differences were caused by insulinopenia and hyperglycemia or other metabolic parameters. The intent was to perform a hypothesis-generating proof-of-concept evaluation of medication-specific behavioral change among individuals who demonstrated early weight-loss response, thereby reducing noise from early discontinuation and incomplete follow-up commonly observed in routine care.

#### 2.3.3. Data Collection and Variables

Demographic and clinical variables recorded at baseline included sex, age, self-reported duration of obesity, height, baseline and six months weight, baseline and six months abdominal circumference, and concomitant use of antidepressants (yes/no). Laboratory measures obtained as part of standard care were extracted at baseline and follow-up when available, including fasting plasma glucose, HbA1c, total cholesterol, HDL-c, LDL-c, VLDL-c, and triglycerides.

Data collection was performed using medical records, which were entered into a REDCap database. From this database, we extracted a table with all variables and the two EFCA scores from the initial medical consultations (time zero) and the third medical consultations for all participants (time six months). Each medical consultation was conducted three months apart, and appointments were scheduled directly by resident physicians according to our outpatient service’s schedule; therefore, there was only a small delay of one or two weeks, ensuring that the weight comparison was not too discrepant. Patients with missing weight or EFCA data were excluded from the analysis, and missing data from paired fasting plasma glucose, HbA1c, total cholesterol, HDL-c, LDL-c, VLDL-c, and triglycerides were lower than 10%.

#### 2.3.4. Behavioral Phenotyping (EFCA)

Eating behavior was assessed using the Validated EFCA version for Brazilian Portuguese [6], recorded at baseline and 6 months, and their scores were compared. The EFCA yields a total score and domain/subscale scores representing five behavioral dimensions: Hedonic, Emotional, Compulsive, Hyperphagic, and Disorganized eating patterns. Its application is easy and inexpensive, and using a psychometric scale is aligned with the development of obesity as its genesis comes from the central nervous system [8,22]. Considering that Orlistat does not present direct influence in the central nervous system, [23,24] it was not considered in this analysis. A free translation from the validated scale is presented for informative purposes only, as there is no validation for the English language until now. Details from each subscale are presented in Table 2.

English Version—for informative purposes only, not validated

1. I eat until I’m very full.

2. I calm my emotions with food.

3. I ask for more food when I finish my plate.

4. I have a habit of snacking (snacking = having small meals between main meals—breakfast, lunch, afternoon snack, and dinner—without measuring the amount of food eaten).

5. When I start eating something I really like, I have difficulty stopping.

6. I usually eat more than one dish at main meals.

7. I snack between meals due to anxiety, boredom, loneliness, fear, anger, sadness, and/or tiredness.

8. I feel tempted to eat when I see/smell food I like and/or when I pass by a kiosk, a bakery, a pizzeria, or a fast-food establishment.

9. I eat breakfast every day.

10. I eat when I am: bored, anxious, nervous, sad, tired, irritated, and lonely.

11. I skip some—or at least one—of the main meals (breakfast, lunch, afternoon snack, or dinner).

12. When I come across food that I really like, even without feeling hungry, I end up eating it.

13. I eat a lot of food in a short amount of time.

14. When I eat something I like, I finish the whole portion.

15. When I eat something I really like, I eat very quickly.

16. I go more than 5 h a day without eating.

### 2.4. Anthropometric Outcomes

Weight change outcomes were defined as the percent of weight loss in comparison to baseline weight. For interpretability, percent weight loss was also expressed as a positive value, so that higher values correspond to greater loss.

### 2.5. Statistical Analysis

Analyses were performed in RStudio V2024.12.1 Build 563 (Posit, Boston, MA, USA), with two-sided tests and α = 0.05. Continuous variables were summarized as mean ± SD, and categorical variables as counts (%). Missingness was handled by available-case analysis for each endpoint.

### 2.6. Within-Medication Change and Effect Size (Cohen’s dz)

To quantify the magnitude of EFCA change within each medication group, we computed Cohen’s dz for paired data for each subscale and for the EFCA total score with 95% confidence intervals. These effect sizes were visualized as a heatmap to facilitate cross-domain comparison across medications. Significance of within-medication change was evaluated using paired t-tests and displayed as conventional significance markers (*p* < 0.05; *p* < 0.01; *p* < 0.001).

### 2.7. Weight Loss Models and Association with Behavioral Change

Percent weight loss was available as a single observation per participant over the interval; weight-loss analyses used standard linear models. We examined whether behavioral change, described as EFCA subscale differences, predicted weight loss while accounting for the AOM group, interpreting these as hypothesis-generating.

## 3. Results

### 3.1. Cohort Overview and Analytic Approach

In this real-world dataset, participants underwent behavioral phenotyping using the *Escala de Fenótipos de Comportamento Alimentar* (EFCA) and were followed pre- to post-treatment across five commonly used anti-obesity pharmacotherapy strategies (semaglutide, naltrexone/bupropion, sibutramine, topiramate, and tirzepatide). Drug-specific behavioral impact was quantified by within-subject change in EFCA subscales (Hedonic, Hyperphagic, Emotional, Compulsive, and Disorganized), using paired comparisons and standardized effect sizes (Cohen’s dz) with 95% confidence intervals; conventional thresholds were adopted (≈0.2 small, 0.5 moderate, 0.8 large). 

### 3.2. Weight Loss Benchmarking Across Pharmacotherapies

Observed mean weight loss magnitudes in the cohort were substantial across medication classes, ranging from ~15% to ~18.0%, with sibutramine showing the highest observed average reduction (17.7%) and tirzepatide averaging 16.7% in this dataset. These values were compared with reference benchmarks from pivotal trials/summary [10,11,12,13,14,15,16,17,18,19] estimates (e.g., 14.9% for semaglutide; 8.54% for naltrexone/bupropion; 11.6% for sibutramine; 6.3% for topiramate; 19.7% for tirzepatide) to contextualize the real-world performance of these subjects. 

### 3.3. Cardiometabolic Response Among Responders

Beyond weight loss, clinically meaningful improvements were detected in anthropometric and metabolic markers. Waist circumference decreased from a mean of 114.9 cm to 105.0 cm (mean paired reduction ≈ 9.91 cm, *p* < 0.001), indicating robust central adiposity improvement. 

For lipids, triglycerides fell from a mean of 122.5 mg/dL to 107.6 mg/dL (mean paired reduction ≈ 17.8 mg/dL, *p* = 0.009). HbA1c fell from a mean of 5.41% to 5.24% (*p* < 0.001), and fasting plasma glucose fell from a mean of 90.3 mg/dL to 85.8 mg/dL (*p* = 0.002).

In contrast, changes in cholesterol were not statistically significant in these paired comparisons, consistent with heterogeneity in baseline cardiometabolic risk and concomitant therapies. These data indicate that the subjects in the research presented an above-average result, and could be seen as a model for evaluating obesity interventions. Table 3 presents the complete clinical outcomes per AOM.

### 3.4. EFCA Subscale Changes Show Distinct “Behavioral Pharmacodynamics”

Within each pharmacotherapy subgroup, EFCA subscales shifted in patterned, medication-specific ways, suggesting that these drugs do not merely reduce weight, but they reduce different drivers of overeating.

**Figure 1:**
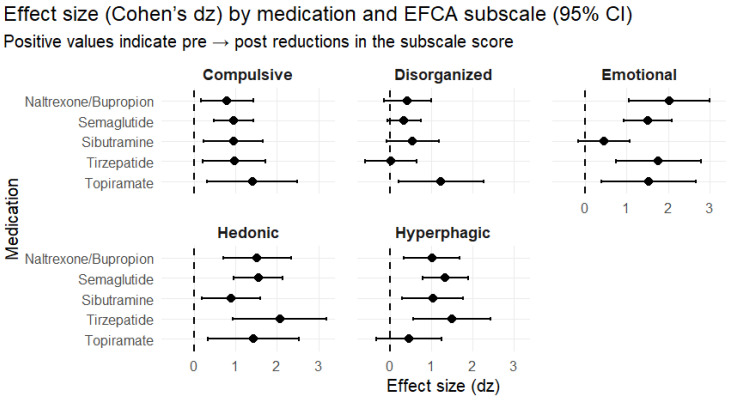
Histogram depicting EFCA subscales reduction for each AOM. The dots stand for Cohen’s dz and the bars stand for each 95% confidence interval.

**Figure 2:**
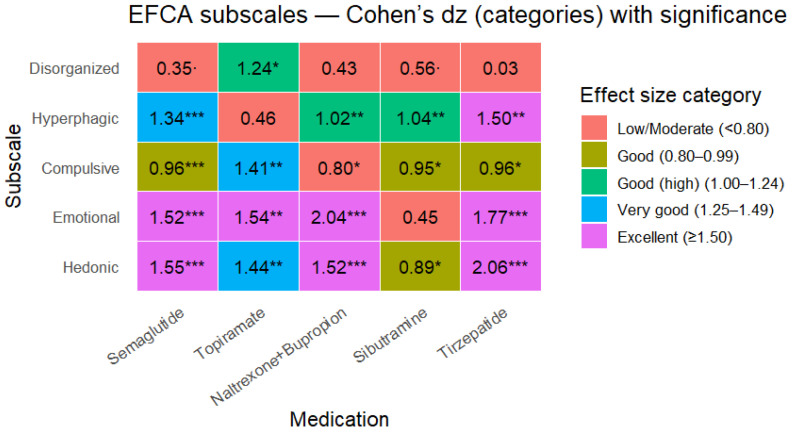
Heat map for each EFCA subscale and AOM. Numbers are Cohen’s dz. (* *p* < 0.05; ** *p* < 0.01; *** *p* < 0.001).

Effect size mapping (Cohen’s dz) across EFCA subscales demonstrated large, selective improvements (many dz ≥ 0.8), with the strongest signals concentrated in hedonic, emotional, hyperphagic, and compulsive domains, while disorganization showed more variable responsiveness (Figure 1 and Figure 2).

Key high-magnitude examples (dz; 95% CI; *p*):

- Emotional eating: naltrexone/bupropion dz 2.04 (1.06–3.01), *p* < 0.001; tirzepatide dz 1.77 (0.74–2.80), *p* < 0.001; semaglutide dz 1.52 (0.94–2.10), *p* < 0.001; topiramate dz 1.54 (0.40–2.68), *p* = 0.0066;
- Hedonic eating: tirzepatide dz 2.06 (0.93–3.20), *p* < 0.001; semaglutide dz 1.55 (0.96–2.13), *p* < 0.001; naltrexone/bupropion dz 1.52 (0.70–2.34), *p* < 0.001; topiramate dz 1.44 (0.34–2.53), *p* = 0.009;
- Hyperphagic pattern: tirzepatide dz 1.50 (0.57–2.43), *p* = 0.001; semaglutide dz 1.34 (0.80–1.89), *p* < 0.001; sibutramine dz 1.04 (0.29–1.78), *p* = 0.006; naltrexone/bupropion dz 1.02 (0.34–1.70), *p* = 0.003;
- Compulsive pattern: topiramate dz 1.41 (0.32–2.49), *p* = 0.0099; tirzepatide dz 0.96 (0.20–1.73), *p* = 0.014; semaglutide dz 0.96 (0.48–1.44), *p* < 0.001; sibutramine dz 0.95 (0.22–1.67), *p* = 0.011;
- Disorganized eating: effects were inconsistent; topiramate showed a large dz (1.24) but with a wide CI, while tirzepatide was essentially neutral (dz 0.03; *p* = 0.93).

Given that analyses were performed in a responder-enriched sample, we adopted more stringent effect size thresholds for interpretability and prioritization of clinically meaningful behavioral changes (good: dz ≥ 0.80; good (high): dz ≥ 1.0; very good: dz ≥ 1.25; excellent ≥ 1.50), while reporting continuous dz estimates and confidence intervals for transparency.

## 4. Discussion

The central novelty of this work is the operationalization of a precision framework for obesity care: instead of evaluating medications solely by kilograms lost, the analysis produces a behavioral response fingerprint—a quantitative map of which appetitive/behavioral drivers (hedonic, emotional, hyperphagic, compulsive, disorganized) improve with which drug class, expressed in standardized effect sizes with confidence intervals. 

Conceptually, this suggests a reframing in AOM pharmacotherapy as targeted treatment of behavioral endophenotypes, enabling a “treat-to-phenotype” strategy: individuals with obesity could receive a high-efficacy AOM, as the Brazilian guideline [4], Canadian guideline [25], and Irish guideline [26], and when it is not available, the choice may be matched to agents showing the largest effects in these domains (e.g., naltrexone/bupropion for higher hedonic/emotional scores), and domains with inconsistent change (e.g., disorganization) become explicit flags for adjunctive behavioral interventions rather than silent causes of nonresponse.

In obesity management, there is always the objective to reach the highest weight loss and above-average weight loss, as unveiled by clinical literature. However, even with high-potency AOM, such as Tirzepatide and Semaglutide, nearly 50% of the people using incretins discontinue their obesity treatment due to insurance-related issues [27]. Therefore, considering obesity as the chronic condition it is, EFCA could be used not only to help in choosing the first medication if one is unable to use incretins, but also for tapering the medication after reaching a significant weight loss [28] or for its exchange due to adverse symptoms or insurance-related issues.

Topiramate was included as it is an off-label medication for obesity management in Brazil, as it is an affordable choice and it is often combined with Sertraline or Fluoxetine for “low cost” options. There are combinations of Topiramate with Phentermine [29], and there is evidence that its combination with Sibutramine provides an interesting result [30]; thus, we chose to analyze its use.

While Semaglutide and Tirzepatide are widely recognized as the most potent AOM available to date, our sample presents some limitations. First of all, not all Tirzepatide doses were available in Brazil, and its maximum dose was 5 mg/week [19]. Second, among Semaglutide users, we had people using the subcutaneous version and oral version, in doses ranging from 0.25 mg/week to 2.4 mg/week (subcutaneous) to 3 mg/day to 14 mg/day (oral) [10,11,12]. Thus, their results may be underestimated.

In practical terms, EFCA use suggests a guided prescription that could transform obesity management from a weight-centric endpoint into a psychometric mechanism-informed clinical workflow, where the clinician can justify AOM selection and sequencing based on measurable, person-specific drivers of overeating—and then verify response using the same behavioral metrics longitudinally.

## 5. Conclusions

In this real-world cohort of patients with obesity undergoing pharmacological treatment, clinically meaningful weight loss was accompanied by significant reductions in EFCA total score and multiple eating behavior subscales, with distinct response patterns across medications. While overall weight loss did not consistently differ between treatments in adjusted models, the magnitude and profile of behavioral change varied by drug class, suggesting that anti-obesity medications may exert differentiated effects on specific eating behavior dimensions.

A key finding of this study is the use of within-treatment effect size mapping (Cohen’s dz) to characterize medication-specific behavioral signatures across EFCA subscales. This approach revealed that some medications were associated with stronger effects in hedonic and emotional domains, whereas others showed more prominent effects in compulsive or hyperphagic dimensions. These patterns support the hypothesis that phenotypic characterization of eating behavior may help refine treatment selection beyond weight loss alone.

Importantly, this study advances a behaviorally informed framework for precision obesity pharmacotherapy in routine clinical practice. Rather than treating eating behavior phenotypes as descriptive labels only, our findings suggest they may function as actionable clinical targets and potential moderators of treatment response. This is particularly relevant to nutritional sciences, where behavioral drivers of intake are central determinants of long-term energy balance, adherence, and metabolic outcomes.

The present results should be interpreted in light of the study’s limitations, including the retrospective design, modest sample size in some treatment strata, missing data, lack of randomization, and potential confounding by indication and concomitant therapies. Therefore, causality cannot be inferred, and these findings should be considered hypothesis-generating.

These findings, however, suggest a shift from a “one-size-fits-all” pharmacological approach toward a phenotype-responsive model of obesity care, in which behavioral profiling helps identify the most biologically and behaviorally coherent treatment strategy for each patient.

Nonetheless, this study provides an original and clinically relevant signal: obesity medications may differ not only in the amount of weight loss achieved, but also in the behavioral pathways through which that weight loss is facilitated. Prospective, adequately powered studies are warranted to validate EFCA-based phenotyping as a tool for treatment matching and to test whether phenotype-guided pharmacotherapy improves weight loss durability, adherence, and metabolic health.

## Figures and Tables

**Table 1:** Baseline of all individuals per AOM.

AOM	Overall	Naltrexone/ Bupropione	Semaglutide	Sibutramine	Tirzepatide	Topiramate	*p*-Value ^1^
Number, %	66 (100%)	13 (19.7%)	25 (37.8%)	11 (16.7%)	10 (15.1%)	7 (10.6%)	
Men, %	13 (20%)	2 (15.4%)	6 (24%)	2 (18.2%)	2 (20%)	1 (14.3%)	>0.9
Women, %	53 (80%)	11 (84.6)	19 (76%)	9 (81.8%)	8 (80%)	6 (85.7%)	
Age, in years	44 ± 11	48.4 ± 9.1	43.0 ± 10.4	42.0 ± 14.1	42.3 ± 9.6	45.3 ± 9.4	0.6
Weight, in kg	106 ± 26	106.0 ± 28.3	105.2 ± 24.1	110.3 ± 23.8	111.0 ± 32.4	98.7 ± 22.2	>0.9
BMI, in kg/m^2^	40 ± 8	39.0 ± 10.0	39.2 ± 8.0	42.4 ± 7.5	39.3 ± 7.5	39.7 ± 7.8	0.7
Waist, in cm	115 ± 17	113.6 ± 16.0	115.7 ± 16.6	118.4 ± 17.3	111.3 ± 22.0	113.6 ± 15.6	0.8
EFCA, total	51 ± 12	53.5 ± 9.9	51.2 ± 12.9	44.6 ± 8.9	56.6 ± 14.0	50.4 ± 10.0	0.2
Hedonic	14 ± 4	15.1 ± 2.8	14.0 ± 3.6	13.0 ± 4.5	15.6 ± 4.4	13.0 ± 4.4	0.4
Emotional	14 ± 4	15.8 ± 2.7	13.5 ± 3.9	10.0 ± 5.1	16.0 ± 4.5	14.0 ± 4.1	0.018 *
Compulsive	7 ± 3	6.1 ± 2.8	6.3 ± 2.8	5.7 ± 2.6	7.3 ± 3.1	8.3 ± 1.7	0.3
Hyperphagic	9 ± 4	8.3 ± 3.3	9.4 ± 3.6	7.2 ± 3.0	10.2 ± 3.8	7.0 ± 3.2	0.2
Disorganized	8 ± 3	8.2 ± 2.8	8.1 ± 3.8	8.7 ± 3.7	7.5 ± 2.8	8.1 ± 2.9	>0.9
Glucose, mg/dL	90 ± 11	92.4 ± 11.4	90.5 ± 11.0	91.7 ± 10.8	86.2 ± 10.9	88.8 ± 8.8	0.6
HbA1c, %	5.41 ± 0.43	5.42 ± 0.42	5.47 ± 0.42	5.46 ± 0.42	5.25 ± 0.55	5.37 ± 0.39	0.5
LDL-c, mg/dL	113 ± 32	125.5 ± 29.0	109.7 ± 29.4	110.4 ± 42.5	115.8 ± 35.8	98.5 ± 19.5	0.4
HDL-c, mg/dL	49 ± 13	48.1 ± 8.4	50.0 ± 17.8	46.4 ± 7.3	48.5 ± 12.6	49.6 ± 10.1	>0.9
Triglycerides, mg/dL	123 ± 55	109.9 ± 30.8	126.2 ± 67.3	128.4 ± 59.9	122.8 ± 46.1	125.2 ± 58.3	>0.9

^1^ Pearson’s Chi-squared test; Kruskal–Wallis rank sum test for differences among AOM groups. * Significant *p*-value.

**Table 2:** EFCA Subscales Interpretation [5,6].

Subscale	Interpretation
Hedonic	Eating primarily driven by food reward and pleasure (cravings, “wanting” beyond hunger)
Emotional	Eating triggered by emotions and stress as a coping strategy rather than physiological hunger
Compulsive	Recurrent loss-of-control eating with urge-driven episodes and difficulty stopping once started
Hyperphagic	Predominantly hunger/satiety dysregulation with large portions and persistent appetite (high drive to eat)
Disorganized	Irregular, unstructured eating (skipping meals, chaotic timing, poor planning) with inconsistent self-regulation

**Table 3:** Clinical outcomes per AOM.

AOM	Overall	Naltrexone/ Bupropione	Semaglutide	Sibutramine	Tirzepatide	Topiramate	*p*-Value ^1^
Weight loss, %	16.0 ± 5.9	15.4 ± 4.8	15.5 ± 6.7	17.7 ± 4.4	16.7 ± 6.4	15.5 ± 6.9	0.6
Weight loss, kg	16.7 ± 6.1	15.7 ± 4.3	15.8 ± 6.3	19.3 ± 5.4	18.4 ± 8.2	14.8 ± 5.8	0.3
Waist variation, cm	9.9 ± 6.8	11.5 ± 5.4	10.5 ± 6.9	8.2 ± 5.5	10.1 ± 4.1	7.4 ± 12.2	0.7
HbA1c reduction, %	0.23 ± 0.32	0.20 ± 0.26	0.28 ± 0.25	0.17 ± 0.25	0.42 ± 0.61	0.03 ± 0.57	0.7
Glucose reduction, mg/dL	6 ± 12	4.3 ± 12.4	7.9 ± 10.3	3.6 ± 15.6	9.0 ± 17.2	1.8 ± 4.3	0.8
LDL-c reduction, mg/dL	3 ± 29	9.3 ± 27.5	4.8 ± 32.1	7.9 ± 29.7	26.7 ± 11.0	13.0 ± 19.2	0.12
HDL-c increase, mg/dL	0.7 ± 14.5	1.0 ± 5.9	5.0 ± 20.6	3.5 ± 10.8	1.3 ± 8.1	1.2 ± 6.4	0.9
Triglycerides reduction, mg/dL	18 ± 43	2.5 ± 26.8	20.2 ± 37.3	24.2 ± 70.0	19.7 ± 15.0	28.0 ± 33.4	0.6

^1^ Pearson’s Chi-squared test; Kruskal–Wallis rank sum test for differences among AOM groups.

## Data Availability

Research data is stored in Santa Casa de Misericórdia de São Paulo REDCap server (https://redcap.fcmsantacasasp.edu.br/, accessed on 15 April 2026).

## Abbreviations

The following abbreviations are used in this manuscript:

EFCA	Eating Behavior Phenotypical Scale
AOM	Anti-Obesity Medication
HbA1c	Glycated Hemoglobin
HDL-c	High-density cholesterol
LDL-c	Low-density cholesterol
VLDL-c	Very low-density cholesterol
